# Selective preoperative lower extremity computed tomography angiography before mandibular reconstruction: Identifying patients unlikely to require fibula free flap reconstruction

**DOI:** 10.1007/s10006-026-01646-1

**Published:** 2026-10-13

**Authors:** Jannik Ketschau, Jonathan Mohr, Yannik Leonhardt, Alex Grabenhorst, Hannes Singer, Helena Kram, Nils Krautkremer, Minli Niu, Katharina Pippich, Klaus-Dietrich Wolff, Lucas M. Ritschl

**Affiliations:** 1 Department of Oral and Maxillofacial Surgery, Klinikum Rechts Der Isar, School of Medicine and Health, TUM University Hospital, Technical University of Munich, Ismaninger Strasse 22, 81675 Munich, Germany; 2 Institut of Diagnostic and Interventional Radiology, Klinikum Rechts Der Isar, School of Medicine and Health, TUM University Hospital, Technical University of Munich, Munich, Germany

**Keywords:** Mandibular reconstruction, Fibula free flap, Computed tomography angiography, Preoperative imaging, Clinical decision-making, Resource utilization

## Abstract

**Purpose:**

Not every patient undergoing mandibular continuity resection requires free fibula flap (FFF) reconstruction. Whether clinical judgment reliably identifies patients unlikely to benefit from preoperative lower-extremity computed tomography angiography (CTA) remains unknown, despite the relevance of this question for cost-conscious and resource-efficient imaging strategies.

**Methods:**

A retrospective outcome-based validation was conducted in patients undergoing mandibular continuity resection between July 2012 and June 2023. The primary outcome was secondary FFF reconstruction among patients not initially selected for CTA. Factors associated with CTA utilization were assessed by multivariable logistic regression.

**Results:**

316 consecutively operated patients were included. Prior radiotherapy (OR 2.67, *p* = 0.005) and smoking history (OR 1.98, *p* = 0.032) were independently associated with CTA utilization. Among 111 patients without primary FFF reconstruction, four (3.6%) required secondary FFF reconstruction, all of whom had undergone CTA. No patient managed without CTA subsequently required FFF reconstruction.

**Conclusions:**

In this cohort, clinical judgment was associated with successful identification of patients who did not subsequently require fibular reconstruction. Omission of CTA in carefully selected patients was not associated with subsequent reconstructive deficiency, though larger prospective studies are needed to confirm these findings.

**Supplementary Information:**

The online version contains supplementary material available at https://doi.org/10.1007/s10006-026-01646-1.

## Introduction

Microvascular fibular free flap (FFF) reconstruction remains the gold standard for restoration of mandibular continuity defects, offering reliable osseous reconstruction with favorable functional and aesthetic outcomes [1–3]. Because successful FFF harvest depends on adequate lower-extremity vascular anatomy, preoperative computed tomography angiography (CTA) has become an integral component of reconstructive planning, allowing detailed evaluation of vascular anatomy, identification of anatomical variants, and detection of peripheral vascular disease that may preclude or modify fibula harvest [4–7]. Beyond vascular assessment, the resulting DICOM (digital imaging and information in medicine) data-set of the CTA is increasingly used for virtual surgical planning and CAD/CAM (computer-aided desing/computer-aided manufacturing) assisted workflows, including perforator mapping, osteotomy planning, and fabrication of patient-specific cutting guides [3, 8, 9]. Consequently, many centers now perform preoperative CTA routinely when FFF is planned for bony jaw reconstruction.

However, not every patient undergoing mandibular continuity resection ultimately requires FFF reconstruction. The reconstructive strategy is determined by a combination of defect characteristics and localization, oncologic context, prior treatment history, and patient-related factors, and in a relevant proportion of patients, non-osseous reconstruction represents the appropriate primary approach [10]. In daily clinical practice, the decision to obtain CTA is therefore not universal. Instead, surgeons first assess the anticipated reconstructive strategy and obtain CTA only when FFF reconstruction is considered a realistic option. Despite the central role of this initial gatekeeping decision, its validity has never been examined through long-term patient outcomes. Beyond clinical safety, this question carries direct relevance for resource allocation: in many healthcare systems, imaging capacity is limited and subject to increasing cost pressures, making the judicious use of preoperative CTA a matter of practical as well as clinical importance.

The present study was designed as a retrospective, outcome-based validation of this clinical selection process. The primary question was not whether surgeons decided correctly at the time of initial treatment, but whether omission of CTA resulted in patients being undertreated over the observed study period, as evidenced by the subsequent need for secondary fibular reconstruction. We hypothesized that patients not selected for CTA would not subsequently require fibular reconstruction, thereby providing preliminary outcome-based evidence regarding the validity of clinical judgment as a gating criterion for preoperative vascular imaging. In addition, we aimed to identify clinical factors associated with CTA utilization in order to better characterize the decision-making process underlying preoperative imaging in this setting.

## Materials and methods

### Ethics statement and study design

All clinical investigations were conducted in accordance with the Declaration of Helsinki. The study was approved by the institutional ethics committee of the Technical University of Munich, Klinikum rechts der Isar (2023–459-S-SB). Due to the retrospective nature of the study and complete anonymization of all patient data, individual consent to publish was not required.

This cohort study included all patients who underwent mandibular continuity resection between July 2012 and June 2023 at the Department of Oral and Maxillofacial Surgery, TUM University Hospital Rechts der Isar, Technical University of Munich, Germany. Patients were identified through the institutional digital operating room database using procedure-specific codes for mandibular continuity resection. Mandibular continuity resection was defined as any resection resulting in loss of mandibular continuity, regardless of defect extent or location. Marginal resections without disruption of mandibular continuity were excluded.

Patients were categorized according to preoperative CTA utilization and reconstructive strategy into three groups: (1) patients who underwent CTA and received primary fibular reconstruction, (2) patients who underwent CTA but did not receive primary fibular reconstruction, and (3) patients who did not undergo CTA and did not receive primary fibular reconstruction. Reconstructive outcomes were additionally evaluated in the subgroup of patients who did not undergo primary fibular reconstruction, with particular focus on the occurrence of delayed fibular reconstruction as the primary validation endpoint.

Case identification and manual review were performed by two authors independently, with discrepancies resolved by consensus.

### Patient data collection

Clinical data were retrospectively extracted from the institutional hospital information system (SAP). Variables included age, sex, body mass index (BMI), smoking status, alcohol consumption, diabetes mellitus, vascular comorbidities, ASA classification (American Society of Anesthesiologists physical status), previous head and neck operations, previous reconstructive attempts, preoperative radiotherapy, and surgical indication. Reconstructive variables included the type of primary reconstruction, performance of FFF reconstruction, and the occurrence of subsequent reconstructive procedures. For patients without primary FFF reconstruction, follow-up data were reviewed to identify secondary reconstructive interventions, including delayed/secondary FFF reconstruction, revision surgery, and additional reconstructive procedures. Due to the retrospective study design, missing data were present for selected variables. Analyses were therefore performed using available-case methodology, and the number of evaluable cases is reported where applicable.

### Outcome measures

The primary outcome of this study was the rate of delayed (secondary) FFF reconstruction among patients not selected for preoperative CTA. This endpoint was chosen as an objective measure of the validity of the clinical selection process. The underlying rationale is as follows: if clinical judgment reliably identifies patients unlikely to require FFF reconstruction, secondary fibular reconstruction should be absent or exceedingly rare among patients in whom CTA was omitted. Conversely, if secondary fibular reconstruction occurs at a meaningful rate in this group, this would indicate that patients were initially undertreated as a consequence of inappropriate CTA omission and inadequate reconstructive planning. By anchoring the primary outcome to long-term reconstructive need rather than to the initial clinical decision itself, this design avoids the tautological interpretation that patients managed without CTA did not need CTA simply because they were selected not to receive it.

Secondary outcomes included the clinical factors independently associated with preoperative CTA utilization, characterizing the patient profile considered to warrant preoperative vascular imaging. Further, secondary outcomes comprised the rate of any subsequent reconstructive procedure, the number of subsequent reconstructive interventions, and the type of first subsequent reconstruction among patients who did not undergo primary fibular reconstruction. Clinical characteristics of patients ultimately requiring secondary fibular reconstruction were analyzed descriptively.

### Statistical analysis

Categorical variables are presented as frequencies and percentages and were compared using Pearson's chi-square test or Fisher's exact test, as appropriate. Continuous variables were assessed for normality using visual inspection of histograms and Q–Q plots. Non-normally distributed variables are reported as median and interquartile range (IQR) or range and were compared using Mann–Whitney U tests or Kruskal–Wallis tests, as appropriate.

To identify factors independently associated with CTA utilization, multivariable binary logistic regression analysis was performed. Only variables available prior to CTA decision-making and reconstructive planning were included in the model. Candidate variables were selected a priori based on clinical plausibility and their potential relevance to the preoperative CTA decision. The following variables were included in the multivariable model: age, sex, smoking history, prior radiotherapy, ASA classification, and vascular comorbidity. Odds ratios (ORs) with corresponding 95% confidence intervals (CI) were calculated. Model calibration was assessed using the Hosmer–Lemeshow goodness-of-fit test. No adjustment for multiple testing was applied, as all comparisons were considered exploratory in nature. Exploratory analyses of factors associated with primary fibular reconstruction are provided in the supplementary material. Statistical significance was defined as *p* < 0.05. All analyses were performed using IBM SPSS Statistics (Version 29).

## Results

### Study cohort

**Fig. 1:**
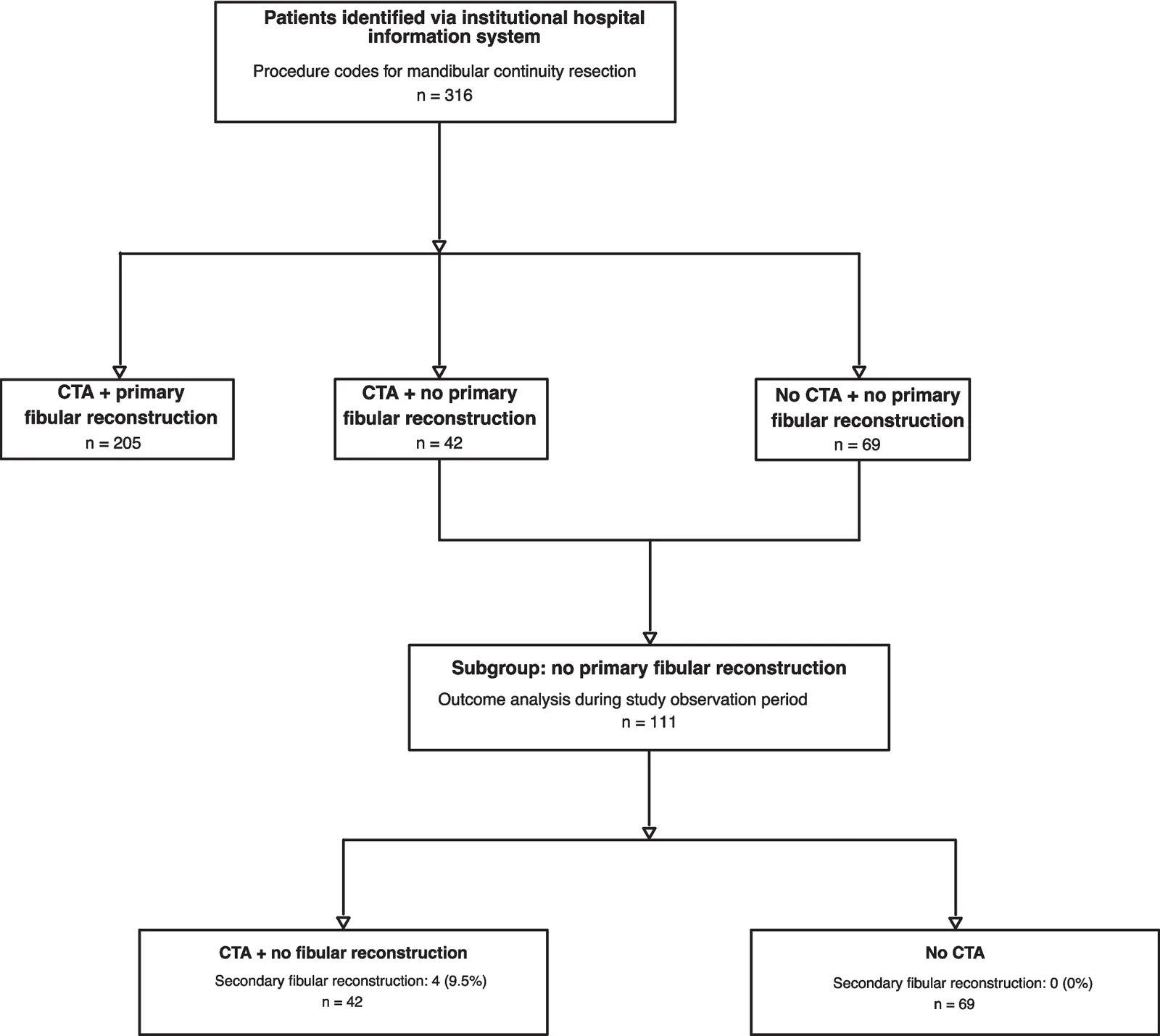
Study flowchart. Flowchart illustrating patient selection and group allocation. All patients who underwent mandibular continuity resection between July 2012 and June 2023 were categorized according to preoperative computed tomography angiography (CTA) utilization and primary reconstructive strategy. The subgroup of patients without primary fibular reconstruction was further analyzed with respect to reconstructive outcomes during the study observation period, with particular focus on the occurrence of salvage fibular reconstruction

A total of 316 patients met the inclusion criteria. These patients were categorized into three groups according to CTA utilization and reconstructive strategy: CTA with primary fibular reconstruction (n = 205), CTA without primary fibular reconstruction (n = 42), and no CTA without primary fibular reconstruction (n = 69) (Fig. 1). Baseline characteristics are summarized in Table 1.

**Table 1:** Baseline characteristics according to CTA/Fibula group. Baseline characteristics of patients undergoing mandibular continuity resection stratified by preoperative lower extremity CTA and primary fibula reconstruction status. Continuous variables are presented as median (interquartile range) or mean ± standard deviation, as appropriate. Categorical variables are presented as number (percentage). Percentages are based on available cases; not all clinical variables were completely documented for every patient due to the retrospective study design. Statistical significant results are shown in bold

Variable	CTA + Fibula (n = 205)	CTA + no Fibula (n = 42)	No CTA (n = 69)	*p*-value
Age (years)	61 (53–69)	68 (58–74)	65 (54.5–73.5)	**0.012**
BMI (kg/m^2^)	23.4 (20.8–25.8)	23.5 (21.5–25.2)	24.5 (21.6–26.4)	0.376
Previous head and neck operations	1 (0–6)	0.00 (0–2)	0.00 (0–3)	**0.009**
Previous reconstructive attempts	0.00 (0–5)	0.00 (0–2)	0.00 (0–2)	**0.022**
Sex (male)	138 (63.9%)	29 (69.0%)	38 (55.1%)	0.277
Prior radiotherapy	92 (42.6%)	12 (28.6%)	12 (17.4%)	** < 0.001**
Previous bony reconstruction	13 (6.0%)	1 (2.4%)	0 (0%)	0.080
ASA classification				
* ASA I*	15 (8.2%)	3 (8.3%)	11 (18.0%)	
* ASA II*	102 (55.4%)	17 (47.2%)	24 (39.3%)	
* ASA III*	65 (35.3%)	16 (44.4%)	26 (42.6%)	
* ASA IV*	2 (1.1%)	0 (0%)	0 (0%)	0.170
Diabetes				
* No*	167 (92.3%)	29 (78.4%)	52 (91.2%)	
* Yes*	14 (7.7%)	8 (21.6%)	5 (8.8%)	**0.034**
Cardiovascular Comorbidity				
* No*	181 (88.3%)	32 (76.2%)	60 (87.0%)	
* Yes*	24 (11.1%)	10 (23.8%)	9 (13.0%)	0.201
Smoking				
* No*	98 (54.4%)	20 (57.1%)	39 (68.4%)	
* Yes*	82 (45.6%)	15 (42.9%)	18 (31.6%)	0.176
Alcohol				
* No*	129 (70.9%)	20 (58.8%)	42 (72.4%)	
* Yes*	53 (29.1%)	14 (41.2%)	16 (27.6%)	0.328
Entity				
* OSCC*	88 (42.9%)	27 (64.3%)	29 (42.0%)	
* ORN*	59 (28.7%)	7 (16.7%)	7 (10.1%)	
* MRONJ/BRONJ*	13 (6.3%)	1 (2.4%)	3 (4.3%)	
* Osteomyelitis*	6 (2.9%)	1 (2.4%)	3 (4.3%)	
* Other tumor entity*	15 (7.3%)	5 (11.9%)	21 (30.4%)	
* Wound healing disorder*	16 (7.8%)	0 (0%)	1 (1.4%)	
Other*	8 (3.9%)	1 (2.4%)	5 (7.2%)	** < 0.001**
Defect localization				0.100
* Anterior/central mandible*	110 (53.7%)	15 (35.7%)	33 (47.8%)	
* Posterior/lateral mandible (incl. angulus)*	55 (26.8%)	13 (30.9%)	19 (27.5%)	
* Combined/other*	40 (19.5%)	14 (33.3%)	17 (24.6%)	
**Abbreviations:** BMI = body mass index; CTA = computed tomography angiography; ASA = American Society of Anesthesiologists physical status classification; OSCC = oral squamous cell carcinoma; ORN = osteoradionecrosis; MRONJ = medication-related osteonecrosis of the jaw; BRONJ = bisphosphonate-related osteonecrosis of the jaw *Other indications included trauma, cystic lesions, vascular malformations, and lesions of unclear etiology				

Significant differences between groups were observed for age (*p* = 0.012), previous head and neck operations (*p* = 0.009), previous reconstructive attempts (*p* = 0.022), positive history of prior radiotherapy (*p* < 0.001), diabetes (*p* = 0.034) and surgical indication (*p* < 0.001). Patients undergoing CTA and primary fibular reconstruction were younger and more frequently had a history of prior radiotherapy and previous reconstructive procedures. No significant differences were observed regarding sex, BMI, smoking status, alcohol consumption, ASA classification, cardiovascular comorbidities, or defect localization (Table 1).

### Factors associated with CTA utilization

To characterize the clinical selection process for preoperative CTA, a multivariable logistic regression analysis was performed including age, sex, smoking history, prior radiotherapy, ASA status, and vascular comorbidity (Table 2). The model was based on 267 of 316 patients with complete data for all included variables; 49 patients (15.5%) were excluded due to missing values in at least one model variable. Prior radiotherapy (OR 2.67, 95% CI 1.34–5.31; *p* = 0.005) and smoking history (OR 1.98, 95% CI 1.06–3.71; *p* = 0.032) were independently associated with CTA utilization. Localization, age, sex, ASA status, and cardiovascular comorbidity were not significantly associated with CTA use.

**Table 2:** Multivariable logistic regression analysis of factors associated with preoperative CTA utilization. Multivariable binary logistic regression model evaluating clinical factors associated with the use of preoperative lower-extremity computed tomography angiography (CTA) prior to mandibular continuity resection. The model was based on 267 patients with complete data (49 patients excluded due to missing values in at least one model variable). Odds ratios (ORs) are presented with 95% confidence intervals (CIs). Statistical significant results are shown in bold

Variable	OR	95% CI	*p*-value
Age	1.00	0.98–1.03	0.700
Male sex	1.38	0.76–2.52	0.289
Smoking history	1.98	1.06–3.71	**0.032**
Prior radiotherapy	2.67	1.34–5.31	**0.005**
ASA ≥ 3	0.59	0.31–1.10	0.094
Vascular comorbidity	1.42	0.57–3.51	0.449
**Model performance:** Omnibus test p < 0.001; Nagelkerke R^2^ = 0.103; Hosmer–Lemeshow *p* = 0.641			

Exploratory univariable analyses and a separate multivariable model investigating factors associated with primary fibular reconstruction are provided in the supplementary material (**supplementary appendix **Tables S1**–S3**). Across these analyses, prior radiotherapy consistently emerged as the strongest factor associated with both CTA utilization and fibular reconstruction.

### Long-term outcomes of patients without primary free fibula flap reconstruction

Reconstructive outcomes during the study observation period were evaluated in the 111 patients who did not undergo primary FFF reconstruction (42 CTA patients and 69 non-CTA patients; Fig. 1 and Table 3).

**Table 3:** Observed Outcomes of patients without primary free fibula flap reconstruction. Comparison of patients who underwent preoperative lower-extremity computed tomography angiography (CTA) but did not receive primary fibular reconstruction and patients managed without CTA and without primary fibular reconstruction. Data are presented as n (%) or median (range). Secondary fibula reconstruction was defined as delayed secondary fibular free flap reconstruction performed after the initial mandibular reconstruction. Statistical significant results are shown in bold

Outcome	CTA + no FFF (n = 42)	No CTA (n = 69)	*p*-value
Any subsequent reconstruction	9 (21.4%)	8 (11.6%)	0.183
Number of subsequent reconstructions	0 (0–2)	0 (0–1)	0.089
Secondary fibula reconstruction	4 (9.5%)	0 (0%)	**0.019**
First subsequent reconstruction: soft tissue flap	2 (4.8%)	6 (8.7%)	
First subsequent reconstruction: plate-based reconstruction	3 (7.1%)	1 (1.4%)	
First subsequent reconstruction: bony reconstruction	4 (9.5%)	1 (1.4%)	0.067
No subsequent reconstruction	33 (78.6%)	61 (88.4%)	0.072

Overall, 17 patients (15.3%) required at least one subsequent reconstructive procedure during the study observation period. Additional reconstructive procedures were observed in 9 of 42 patients (21.4%) in the CTA cohort and in 8 of 69 patients (11.6%) in the non-CTA cohort, although this difference did not reach statistical significance (*p* = 0.183).

Patients in the CTA cohort tended to undergo a greater number of subsequent reconstructive procedures than patients without CTA (*p* = 0.089). Furthermore, the distribution of the first subsequent reconstructive procedure differed between groups without reaching statistical significance (*p* = 0.072). Soft-tissue flap reconstruction represented the most common secondary procedure in the non-CTA cohort, whereas plate-based and non-FFF bony reconstructions were more frequently observed among CTA patients.

### Secondary free fibula flap reconstruction

Only four of 111 patients (3.6%) who did not receive primary FFF reconstruction subsequently underwent secondary FFF reconstruction (Table 3). Importantly, all four secondary FFF procedures occurred in patients who had undergone preoperative CTA, whereas no patient in the non-CTA cohort subsequently required FFF (9.5% vs. 0%, *p* = 0.019). When considering any secondary bony reconstruction more broadly, four of 42 CTA patients (9.5%) and one of 69 non-CTA patients (1.4%) ultimately underwent bony reconstruction.

Three of the four patients requiring secondary FFF reconstruction had OSCC, while one patient had an adenocarcinoma of the mandible. All four patients received radiotherapy during the course of treatment. Initial reconstruction consisted of plate reconstruction combined with an anterolateral thigh flap in three patients and an osteomyocutaneous scapular flap in one patient. Two patients had undergone previous head and neck surgery before mandibular continuity resection (Table 4).

**Table 4:** Characteristics of patients requiring secondary free fibula flap reconstruction. Clinical characteristics of the four patients who underwent delayed secondary fibular free flap reconstruction after omission of primary fibular reconstruction

Case	Diagnosis	Prior RT	Adj. RT	Prior H&N surgery	Initial reconstruction
1	OSCC	Yes	Yes	Yes	Plate + ALT-Flap
2	OSCC	Yes	Yes	No	Plate + ALT-Flap
3	OSCC	No	Yes	Yes	Plate + ALT-Flap
4	Adenocarcinoma	No	Yes	No	Scapular flap
**Abbreviations:** OSCC = oral squamous cell carcinoma; RT = radiotherapy; H&N surgery = head and neck surgery; ALT-Flap = anterolateral thigh flap					

No patient managed without CTA subsequently required secondary fibular reconstruction during the study observation period (0 of 69; 95% CI 0.0–5.2%, exact binomial). While this finding is clinically reassuring, the upper bound of the confidence interval indicates that a true underlying event rate of up to approximately 5% cannot be excluded based on the present sample size.

## Discussion

The present study evaluated the validity of clinical judgment as a gating criterion for preoperative lower-extremity CTA in patients undergoing mandibular continuity resection. The principal finding was that no patient managed without preoperative CTA subsequently required FFF reconstruction during the observed period. This finding should not be interpreted as a tautology arising from the retrospective study design. Rather, it constitutes an outcome-based validation of a clinical decision process: the relevant question is not whether surgeons decided correctly at the time of initial treatment, but whether their decisions resulted in patients being undertreated over the observed period. The complete absence of secondary fibular reconstruction in the non-CTA cohort addresses this question and provides preliminary evidence that, within the present institutional cohort, experienced clinical assessment was associated with successful identification of patients who did not subsequently require FFF reconstruction. Beyond diagnostic validity, this finding highlights the importance of judicious resource allocation. In the setting of limited imaging capacity and increasing cost pressures, selective use of preoperative CTA based on clinical judgment avoids unnecessary investigations without compromising patient outcomes. This supports a value-based approach in which diagnostic resources are reserved for patients most likely to benefit, thereby improving efficiency and prioritizing high-risk cases.

Preoperative lower-extremity CTA is widely regarded as an essential component of FFF planning, and its value once fibular reconstruction is being considered is well established [4, 5]. Previous work from our institution demonstrated that CTA findings influence reconstructive planning and flap selection in a relevant proportion of cases, with vascular findings leading to modification of the operative strategy in a subset of patients [11]. Earlier studies by Disa and Cordeiro [12] and Lutz et al. [13] supported a selective imaging strategy based on clinical examination and pedal pulse assessment, reporting excellent flap outcomes and no donor-site ischemic complications when CTA was reserved for patients with abnormal vascular findings. More recent investigations have challenged selective imaging strategies: Ma et al. [14] demonstrated that clinically significant vascular anomalies frequently remain undetected despite normal physical examination findings, while Abou-Foul et al. [15] highlighted the clinical relevance of dominant peroneal artery variants that may be entirely asymptomatic prior to fibula harvest.

These findings have led many authors to advocate routine CTA for all FFF candidates. Importantly, however, the present study addresses a different and logically preceding clinical question: not whether CTA should be performed before FFF harvest, but whether a patient should undergo CTA at all. This distinction is fundamental, as it concerns the initial triage of patients rather than the conduct of fibula flap surgery itself. Our findings do not challenge the role of CTA in FFF planning; they address the step before it.

A distinct subgroup warrants further consideration: patients who underwent preoperative CTA but did not ultimately receive primary FFF reconstruction (n = 42). This group most plausibly represents cases in which FFF reconstruction was considered a realistic preoperative option but was ultimately not performed, due to CTA findings prompting an alternative reconstructive strategy, changes in oncologic circumstances, defect localization or patient-related factors identified during the perioperative course. Notably, this subgroup exhibited the highest rate of subsequent reconstructive procedures and accounted for all four secondary FFF reconstructions observed in the study, consistent with the interpretation that these patients represented the most complex and reconstructively uncertain cases in the entire cohort. Their clinical trajectory further underscores that CTA utilization in this group reflected genuine reconstructive uncertainty rather than institutional routine, and that the absence of CTA in the non-CTA cohort was a considered clinical decision rather than an inadvertent omission.

Computed tomography angiography utilization was independently associated with prior radiotherapy and smoking history, whereas documented vascular comorbidity was not a significant predictor. Moreover, these findings underscore the importance of a comprehensive understanding of the patient's medical history, particularly previous surgical procedures and oncologic treatments. They also highlight the need for patients to be referred for their initial consultation with complete and comprehensive clinical documentation. Especially in times of increasing economic pressure on healthcare systems, this approach may help reduce unnecessary repeat consultations, thereby avoiding redundant resource utilization and duplicated administrative effort. Additionally, this pattern is informative and suggests that CTA utilization was driven by perceived overall reconstructive complexity rather than by isolated concern regarding lower-extremity vascular disease. Prior radiotherapy has consistently been associated with increased reconstructive complexity, higher rates of wound complications, hardware exposure, osteoradionecrosis, and flap-related morbidity [16, 17], and its association with CTA utilization likely reflects the surgeon's anticipation of a challenging reconstructive environment in which FFF reconstruction would be both more necessary and more technically demanding. Smoking is similarly associated with impaired wound healing, compromised tissue perfusion, and increased postoperative complications [18, 19]. Beyond these direct effects, smoking may serve as a proxy for subclinical peripheral vascular disease not yet manifesting as formally documented vascular comorbidity, and surgeons may therefore incorporate smoking history into their assessment of reconstructive complexity and tissue reliability even in the absence of a diagnosed vascular condition. This would explain why smoking, but not documented vascular comorbidity, emerged as an independent predictor of CTA utilization in our model.

It is further noteworthy that, despite the absence of CTA, the non-CTA cohort did not differ significantly from CTA patients in several key markers of surgical risk and general health, including ASA classification, vascular comorbidity, BMI, and smoking status. This argues against the interpretation that the non-CTA group simply represented a universally low-risk or uncomplicated population in whom the absence of subsequent fibular reconstruction was an inevitable and uninteresting finding. Rather, it suggests that the clinical selection process was responsive to reconstructive considerations specific to anticipated defect complexity and treatment history, and that the non-CTA group was not merely a residual category of straightforward cases.

A further potential concern is whether the favorable outcome in the non-CTA cohort was partially attributable to a systematic enrichment of posterior or lateral mandibular defects, which are generally considered more amenable to plate-based reconstruction without subsequent osseous reconstruction (1). Posterior and lateral defects, including isolated molar region and mandibular angle defects, were similarly distributed across all three groups, and defect localization did not differ significantly between groups overall (*p* = 0.100). When comparing the two groups most relevant to the primary outcome, patients who underwent CTA but did not receive primary fibular reconstruction and patients managed without CTA, the proportion of anterior and central mandibular defects was comparable (35.7% vs. 47.8%), as was the proportion of posterior and lateral defects (30.9% vs. 27.5%), providing no evidence of systematic defect localization bias between these groups.

The outcome data further support the validity of the initial selection process. Among 111 patients who did not undergo primary fibular reconstruction, only four ultimately required secondary fibular reconstruction, and all four belonged to the CTA cohort. Three had OSCC, and all four received radiotherapy during the course of treatment, with initial reconstruction consisting of plate-based techniques combined with soft-tissue free flap coverage in three patients and a scapular free flap in one. This clinical profile is consistent with the established literature, in which secondary fibular reconstruction after plate-based or soft-tissue reconstruction is most commonly necessitated by plate exposure, osteoradionecrosis, recurrent disease, and soft-tissue failure [20, 21]. The characteristics of these four patients suggest that their need for secondary reconstruction was driven by challenging oncologic circumstances and treatment-related morbidity rather than by an inappropriate initial reconstructive decision. More broadly, secondary osseous reconstruction may become necessary as a consequence of subsequent disease events, including recurrent malignancy, treatment-related osteoradionecrosis, or late hardware failure following adjuvant radiotherapy, rather than evidence that the initial reconstructive strategy was inappropriate. In this context, secondary fibular reconstruction should be understood as a pragmatic surrogate endpoint for reconstructive adequacy rather than a definitive indicator of initial undertreatment. All four patients who underwent secondary fibular reconstruction received radiotherapy during the course of treatment, suggesting that their need for secondary osseous reconstruction was driven primarily by treatment-related morbidity rather than an inappropriate initial reconstructive decision. Placed in the context of previously reported salvage rates – Wei et al. [20] observed secondary osseous reconstruction in approximately one-third of patients following plate-based reconstruction – the overall rate of 3.6% in the present non-fibula cohort is notably low and consistent with a well-selected patient population.

An important contextual consideration is the increasing integration of virtual surgical planning into contemporary mandibular reconstruction workflows. Modern approaches frequently employ CTA not only for vascular assessment but also for perforator mapping, osteotomy planning, and fabrication of patient-specific cutting guides [3, 8, 9, 22]. In centers routinely employing virtual workflows, the practical distinction between routine and selective CTA may become progressively less relevant, as CTA is effectively required for the planning process regardless of reconstructive uncertainty. Nevertheless, identifying patients unlikely to require fibular reconstruction remains clinically meaningful even in this context, as it may avoid unnecessary imaging and simplify preoperative workup in cases where simpler reconstructive strategies are anticipated from the outset.

### Limitations

The present study has several limitations. Its retrospective single-center design introduces the possibility of selection bias and limits generalizability to other institutions and patient populations. Computed tomography angiography utilization was based on surgeon judgment rather than predefined objective criteria, meaning that the observed associations reflect institutional decision-making patterns that may differ across centers. The number of secondary fibular reconstructions was small, limiting statistical power for detailed subgroup analyses. A prospective study with predefined selection criteria and systematic long-term follow-up would be required to confirm these findings in an independent cohort and to establish formal selection guidelines.

Another relevant limitation is the absence of systematically recorded individual follow-up durations. Given the inclusion period from 2012 to 2023, substantial heterogeneity in observation length can be expected, and the probability of identifying a secondary fibular reconstruction necessarily depends on follow-up completeness. The absence of this event in the non-CTA cohort should therefore be interpreted with appropriate caution, and a prospective study with systematic follow-up documentation would be required to provide more precise long-term estimates. Nevertheless, the consecutive institutional cohort spanning over a decade and the focus on a clinically important yet previously unstudied decision point lend the findings practical relevance for reconstructive surgical planning.

## Conclusion

In conclusion, the present study provides preliminary outcome-based evidence suggesting that clinical judgment may serve as a gating criterion for preoperative lower-extremity CTA in selected patients. No patient who was not selected for CTA subsequently required fibular reconstruction during the study observation period, though a low residual risk cannot be definitively excluded given the sample size. While CTA remains indispensable once fibular reconstruction is being considered, omission of CTA in carefully selected patients was not associated with subsequent reconstructive deficiency in this cohort. Beyond the clinical safety of this approach, selective CTA utilization may meaningfully contribute to more efficient use of imaging resources in everyday reconstructive practice.

## Supplementary Information

Below is the link to the electronic supplementary material.

Supplementary file1 (DOCX 26 KB)

## Data Availability

No datasets were generated or analysed during the current study.

## Abbreviations

ALT: Anterolateral thigh flap
ASA: American Society of Anesthesiologists physical status classification
BMI: Body mass index
BRONJ: Bisphosphonate-related osteonecrosis of the jaw
CAD/CAM: Computer-aided design / computer-aided manufacturing
CI: Confidence interval
CTA: Computed tomography angiography
H&N: Head and neck
IQR: Interquartile range
MRONJ: Medication-related osteonecrosis of the jaw
OR: Odds ratio
ORN: Osteoradionecrosis
OSCC: Oral squamous cell carcinoma
PAD: Peripheral arterial disease
RT: Radiotherapy
VSP: Virtual surgical planning
