# Supplementary material for: Selective preoperative lower extremity computed tomography angiography before mandibular reconstruction: Identifying patients unlikely to require fibula free flap reconstruction
Source: Oral Maxillofac Surg. 2026 Oct 13;30(1):162. doi: 10.1007/s10006-026-01646-1 (PMC13658326; doi:10.1007/s10006-026-01646-1)
Supplement: Supplementary file 1 [file 10006_2026_1646_MOESM1_ESM.docx]

# Selective Preoperative Lower Extremity Computed Tomography Angiography Before Mandibular Reconstruction: Identifying Patients Unlikely to Require Fibula Free Flap Reconstruction

Jannik Ketschau MD ^1^; Jonathan Mohr MD DMD^1^; Yannik Leonhardt MD ^2^; Alex Grabenhorst MD DMD ^1^; Hannes Singer MD DMD ^1^; Helena Kram MD ^1^; Nils Krautkremer MD DMD ^1^; Katharina Pippich MD DMD ^1^; Klaus-Dietrich Wolff MD DMD ^1^; Lucas M. Ritschl MD DMD ^1^

^1^ Department of Oral and Maxillofacial Surgery, TUM University Hospital Klinikum rechts der Isar, School of Medicine and Health, Technical University of Munich, Germany

^2^ Institut of Diagnostic and Interventional Radiology, TUM University Hospital Klinikum rechts der Isar, School of Medicine and Health, Technical University of Munich, Germany

**Appendix**

**Corresponding author:**

Jannik Ketschau MD

Department of Oral and Maxillofacial Surgery

TUM University Hospital Klinikum rechts der Isar

School of Medicine and Health

Technical University of Munich

Ismaninger Strasse 22, D-81675 Munich, Germany

Email: [jannik.ketschau@tum.de](mailto:jannik.ketschau@tum.de)

### Supplementary Table S1. Univariable logistic regression analysis of factors associated with preoperative CTA utilization

| **Variable** | **OR** | **95% CI** | **p-value** |
| --- | --- | --- | --- |
| Age (per year) | 1.00 | 0.98–1.02 | 0.887 |
| Male sex | 1.76 | 1.06–2.92 | 0.027 |
| BMI | 1.01 | 0.95–1.08 | 0.721 |
| Smoking history | 1.92 | 1.07–3.46 | 0.028 |
| Prior radiotherapy | 2.90 | 1.59–5.29 | <0.001 |
| Previous head and neck operations | 1.12 | 0.85–1.48 | 0.430 |
| Previous reconstructive attempts | 1.14 | 0.71–1.84 | 0.601 |
| Diabetes mellitus | 0.91 | 0.37–2.25 | 0.843 |
| ASA ≥3 | 0.75 | 0.44–1.29 | 0.302 |
| Vascular comorbidity | 1.18 | 0.56–2.48 | 0.667 |

Table S1: Univariable binary logistic regression analysis of clinical factors associated with the use of preoperative lower-extremity computed tomography angiography (CTA) prior to mandibular continuity resection. Odds ratios (ORs) are presented with 95% confidence intervals (CIs). Statistically significant results are shown in bold.

Supplementary Table S2*.* Univariable analysis of factors associated with primary fibula reconstruction

| **Variable** | **No primary fibula (n = 111)** | **Primary fibula (n = 220)** | **p-value** |
| --- | --- | --- | --- |
| **Age, years** | Older (mean rank 186.1) | Younger (mean rank 155.9) | **0.007** |
| **BMI, kg/m²** | Mean rank 155.0 | Mean rank 142.9 | 0.244 |
| **Previous head and neck operations** | Mean rank 144.7 | Mean rank 176.7 | **0.002** |
| **Previous reconstructive attempts** | Mean rank 153.2 | Mean rank 172.5 | **0.011** |
| **Male sex** | 67 (60.4%) | 140 (63.6%) | 0.561 |
| **Prior radiotherapy** | 24 (21.6%) | 93 (42.5%) | **<0.001** |
| **Previous bony reconstruction** | 1 (0.9%) | 14 (6.4%) | **0.024** |
| **ASA status** | ASA I: 14.4% ASA II: 42.3% ASA III: 43.3% | ASA I: 10.5% ASA II: 54.3% ASA III: 36.2% | 0.120 |
| **Diabetes mellitus** | 13.8% Type II DM | 6.5% Type II DM | 0.081 |
| **Vascular comorbidity** | 17.1% | 11.4% | 0.294 |
| **Smoking status** | Distribution comparable | Distribution comparable | 0.385 |
| **Alcohol consumption** | Distribution comparable | Distribution comparable | 0.886 |
| **Defect localization** | Distribution differed numerically | Distribution differed numerically | 0.102 |
| **Indication for resection** | Different distribution | Different distribution | **<0.001** |
| **Preoperative reconstructive strategy** | Distribution comparable | Distribution comparable | 0.361 |

Table S2: Univariable analysis of factors associated with primary fibular free flap reconstruction among all patients undergoing mandibular continuity resection (n = 331). Continuous variables are compared using the Mann–Whitney U test and presented as mean ranks. Categorical variables are presented as number (percentage) and compared using Pearson's chi-square test or Fisher's exact test, as appropriate. Statistically significant results are shown in bold.

### Supplementary Table S3. Multivariable logistic regression analysis of factors associated with primary fibula reconstruction

| **Variable** | **OR** | **95% CI** | **p-value** |
| --- | --- | --- | --- |
| Age at surgery (years) | 0.99 | 0.97–1.01 | 0.156 |
| Prior radiotherapy | **2.69** | **1.47–4.92** | **0.001** |
| Previous reconstructive attempts | **1.94** | **1.02–3.69** | **0.044** |
| Vascular comorbidity | 0.64 | 0.30–1.36 | 0.248 |
| ASA III–IV (vs. ASA I–II) | 0.73 | 0.41–1.28 | 0.266 |
| Model performance: Omnibus test p < 0.001; Nagelkerke R² = 0.115; Hosmer–Lemeshow goodness-of-fit test p = 0.493. | | | |

Table S3: Multivariable binary logistic regression model evaluating clinical factors associated with the use of primary fibular free flap reconstruction prior to mandibular continuity resection. Only variables available prior to reconstructive planning were included. Odds ratios (ORs) are presented with 95% confidence intervals (CIs). Statistically significant results are shown in bold.
